# Reference Intervals for Non-Fasting CVD Lipids and Inflammation Markers in Pregnant Indigenous Australian Women

**DOI:** 10.3390/healthcare5040072

**Published:** 2017-10-14

**Authors:** Tracy L. Schumacher, Christopher Oldmeadow, Don Clausen, Loretta Weatherall, Lyniece Keogh, Kirsty G. Pringle, Kym M. Rae

**Affiliations:** 1 Gomeroi gaaynggal Centre, Faculty of Health and Medicine, University of Newcastle, Tamworth, NSW 2340, Australia; tracy.schumacher@newcastle.edu.au (T.L.S.); loretta.weatherall@newcastle.edu.au (L.W.); lyniece.keogh@newcastle.edu.au (L.K.); kirsty.pringle@newcastle.edu.au (K.G.P.); 2 Department of Rural Health, University of Newcastle, Tamworth, NSW 2340, Australia; 3 School of Medicine and Public Health, University of Newcastle, Callaghan, NSW 2308, Australia; christopher.oldmeadow@newcastle.edu.au; 4 Priority Research Centre for Physical Activity and Nutrition, University of Newcastle, Callaghan, NSW 2308, Australia; 5 Public Health Stream, Hunter Medical Research Institute, New Lambton, NSW 2308, Australia; 6 New South Wales Health Pathology, Tamworth, NSW 2340, Australia; don.clausen@hnehealth.nsw.gov.au; 7 School of Biomedical Sciences and Pharmacy, University of Newcastle, Callaghan, NSW 2308, Australia; 8 Priority Research Centre for Reproductive Science, University of Newcastle, Callaghan, NSW 2308, Australia; 9 Priority Research Centre for Generational Health and Ageing, University of Newcastle, Callaghan, NSW 2308, Australia

**Keywords:** cardiovascular, biomarkers, lipids, inflammation, pregnancy, gestation, Australian Aborigine, reference growth curves

## Abstract

Indigenous Australians experience high rates of cardiovascular disease (CVD). The origins of CVD may commence during pregnancy, yet few serum reference values for CVD biomarkers exist specific to the pregnancy period. The Gomeroi gaaynggal research project is a program that undertakes research and provides some health services to pregnant Indigenous women. Three hundred and ninety-nine non-fasting samples provided by the study participants (206 pregnancies and 175 women) have been used to construct reference intervals for CVD biomarkers during this critical time. A pragmatic design was used, in that women were not excluded for the presence of chronic or acute health states. Percentile bands for non-linear relationships were constructed according to the methods of Wright and Royston (2008), using the xriml package in StataIC 13.1. Serum cholesterol, triglycerides, cystatin-C and alkaline phosphatase increased as gestational age progressed, with little change seen in high-sensitivity C-Reactive Protein and γ glutamyl transferase. Values provided in the reference intervals are consistent with findings from other research projects. These reference intervals will form a basis with which future CVD biomarkers for pregnant Indigenous Australian women can be compared.

## 1. Introduction

Cardiovascular disease (CVD) is one of the leading causes of death worldwide, and the most recent comprehensive health survey in Australia highlighted that this is also true for Indigenous Australians [1,2]. The origins of CVD can precede conception, and continue throughout childhood into adulthood [3,4]. Guidelines for assessing CVD risk include obtaining lipid profiles [5], with inflammation markers also providing information on the status of disease progression [6]. Monitoring for these biological markers is not routinely performed during pregnancy, as the levels of these markers change with gestational age, and no specific guidelines in relation to this exist. There is also evidence to suggest that differences in lipid profiles during pregnancy exist due to ethnicity [7]. Lipid and inflammatory biomarker profiles during pregnancy for Indigenous Australian women remain unknown. 

Adverse outcomes for both mother and baby have been linked to elevated lipid levels during pregnancy [8]. Serum triglycerides have been shown to rise 2.7-fold in the third trimester [9], although this has also been associated with increased baby birth weight [8]. Increased triglyceride levels during early pregnancy are associated with pregnancy-induced hypertension, preeclampsia and induced preterm delivery [8], and the presence of gestational diabetes (GDM) has been demonstrated to increase triglyceride levels at this time [10]. Cholesterol profiles change to a lesser extent, with a small decrease being seen in the first trimester and greater changes being seen in the second and third trimesters [9,11]. 

Biomarkers of inflammation, such as high-sensitivity C-Reactive Protein (hs-CRP), may indicate an increased risk of mortality from CVD at any stage of the lifespan [12]. It has also been used in early gestation to indicate an increased risk of preeclampsia [13]. hs-CRP levels are expected to be slightly higher among women who are pregnant, although this is not expected to change over the course of pregnancy [14]. Of the liver enzymes, γ-glutamyl transferase (GGT) and alkaline phosphatase (ALP), have been linked to CVD risk, albeit not during pregnancy [15], with both demonstrating a relationship with hs-CRP [16]. Additionally, cystatin C (cyst-C), a cysteine protease inhibitor, is associated with adverse CVD events and mortality [17]. Levels of cyst-C are expected to rise during the third trimester in pregnancy, likely due to changes in kidney filtration rates [18].

These serum CVD and inflammatory biomarkers have been collected in pregnant Indigenous Australian women as part of their enrollment in the Gomeroi gaaynggal research program in NSW, Australia. Gomeroi gaaynggal means “babies from Gomeroi lands” in the local Kamilaroi/Gomeroi language, and the ongoing program aims to work with local Indigenous communities to improve health outcomes for mothers and their babies. The project was established in 2009, and has provided some health services to pregnant women since the program’s inception. Therefore, the aim is to describe the lipid and inflammation profiles of Indigenous Australian women during mid-late pregnancy as percentile ranges for serum total cholesterol, triglycerides, hs-CRP, ALP, GGT and cyst-C. 

## 2. Materials and Methods

The Gomeroi gaaynggal program is a prospective longitudinal program that follows dyads of Indigenous mothers and their babies from pregnancy until the child is 5 years of age. A full methodology of the program is published elsewhere [19]. Briefly, women are recruited to the study early in their pregnancy via antenatal clinics and birth services based in three recruitment sites in New South Wales (NSW), Australia by Indigenous research staff. These sites originally represented regional, rural and remote communities. Recruitment at the regional site has been discontinued due to difficulties with engagement and recruiting at this location. Study protocols ensure that participants recruited to the study are referred to the appropriate health services if adverse results are detected.

Recruitment for the study began in 2010. Women are eligible to join the program if they identify as being Indigenous, or are carrying an Indigenous baby. Participants may enroll at any stage during their pregnancy, and must provide written informed consent. Ethics approval for this study was provided by the Hunter New England (HNE) Local Health District Human Research Ethics Committee (HREC) (reference number: 08/05/21/4.01), the NSW HREC (reference number: HREC/08/HNE/129, and the Aboriginal Health and Medical Research Council (reference number: 654/08).

Gestational age of the fetus is assessed by ultrasound scan. Approximately three ultrasound scans are scheduled over the course of the pregnancy, with additional scans scheduled if abnormalities or concerns for fetal wellbeing are detected. Ultrasounds are performed using a Phillips Cx50 Portable Diagnostic Ultrasound with a 5 Mhz convex transducer by a qualified sonographer. Scans assess for an estimated due date, multiple pregnancies, and fetal growth, morphology, abnormalities and viability.

The approval for blood sample collection allows for samples to be taken after a gestational age of 84 days, with a total of three samples collected during the pregnancy and taken during ultrasound visits, where possible. Samples were random (non-fasting), and were collected in a 4 mL Vacuette container containing Z serum separator and clot activator by an Indigenous research assistant trained in phlebotomy. Following collection, samples were placed in an insulated container containing ice bricks before being centrifuged and transferred to a NATA (National Association of Testing Authorities) accredited laboratory NSW Health Pathology, Tamworth. The length of time samples were kept in the insulated container between collection and processing varied between 1 and 6 h. Samples were centrifuged for 10 min in either a Hettich Rotofix 32 (Tuttlingen, Germany) at 4000 revolutions per minute (RPM) with a relative centrifugal force (RCF) of 1344 or a Thermofisher Heraeus (Germany) at 3500 RPM (RCF of 1530). Samples were assayed for total cholesterol, triglycerides, hs-CRP, GGT, ALP and cyst-C using standardized pathology protocols in an Abbott Architect Biochemistry Analyser.

Body mass index (BMI) was calculated from self-reported pre-pregnancy weight, and height measured at the participant’s first visit to the Gomeroi gaaynggal centre. Smoking status was obtained at each visit by self-report using an online questionnaire. Pregnancy and birth outcomes were obtained, where possible, from patient files.

### Statistical Analysis

Pregnancies were included for analysis if women had at least one blood sample taken during their pregnancy and the participant was carrying a single fetus only. Variables were tested for linear and non-linear relationships. Percentile bands for non-linear relationships were created according to the methods of Wright and Royston, using the xriml package (Royston and Wright, version 6.0.0 PR, 26 November 2008) in Stata/IC 13.1 for Windows (Stata Corp LP, College Station, TX, USA). This model accounts for non-normal skewness or kurtosis, and estimates percentile curves based on fractional polynomial functions for means and potentially standard deviations (to model heterogeneity of the outcome over time). A fractional polynomial regression of the outcome on time (gestational age) was initially fit to determine the optimal fractional polynomial terms for the mean function. Scaled absolute residuals from this model were then regressed on time to determine the optimal fractional polynomial terms for the standard deviation function. We then estimated the percentile functions assuming normally distributed residuals. This assumption was assessed through quantile plots, histograms and formal goodness of fit tests [20]. When evidence of non-normality was found, the three parameter (an additional parameter for skewness) exponential normal (EN) or the four parameter (an additional kurtosis parameter) modulus exponential normal (MEN) models were estimated, with model fit again assessed with distributional diagnostic plots and formal goodness of fit tests. Two plots and percentile bands were constructed for ALP, based on an age cut-off of 22 years, according to reference ranges for non-pregnant women [21]. Percentile bands for variables showing no relationship with gestation time were generated from the data to match bands presented for non-linear relationships. Data is presented graphically by BMI category and status of adverse pregnancy outcome. 

## 3. Results

A total of 297 women were available for analysis. As many participants re-enrolled in the study for subsequent pregnancies, 343 pregnancies were considered. Pregnancies were included for analysis if women had at least one blood sample taken during their singleton pregnancy. Therefore, 399 blood samples from 206 pregnancies of 175 women were included. Demographics, health characteristics and prior obstetric conditions describing the women at the time of their pregnancy can be found in Table 1. Twenty-nine women did not identify as being Aboriginal or Torres Strait Islander, but identified as carrying a baby of Aboriginal or Torres Strait Islander descent.

Birth outcome data were available for 203 women. A total of 93 women (45.8%) were documented to have had at least one adverse pregnancy outcome for the current pregnancy of interest. Of these, 11 (5.34%) were reported to have had gestational hypertension, 14 (7.6%) preeclampsia, 18 (8.7%) gestational diabetes, 8 (3.9%) proteinuria, 12 (5.8%) albuminuria, 23 (11.2%) large for gestational age and 34 (16.5%) were reported as small for gestational age. 

### Variation in Reference Intervals for Gestational Age

Number of observations that contributed to the figures: cholesterol (*n* = 398); triglycerides (*n* = 397); hs-CRP (*n* = 382); cyst-C (*n* = 348); ALP (*n* = 132 for those aged 16 years to less than 22 years, and *n* = 263 for those aged 22 years and older); and GGT (*n* = 397). 

**Figure 1:**
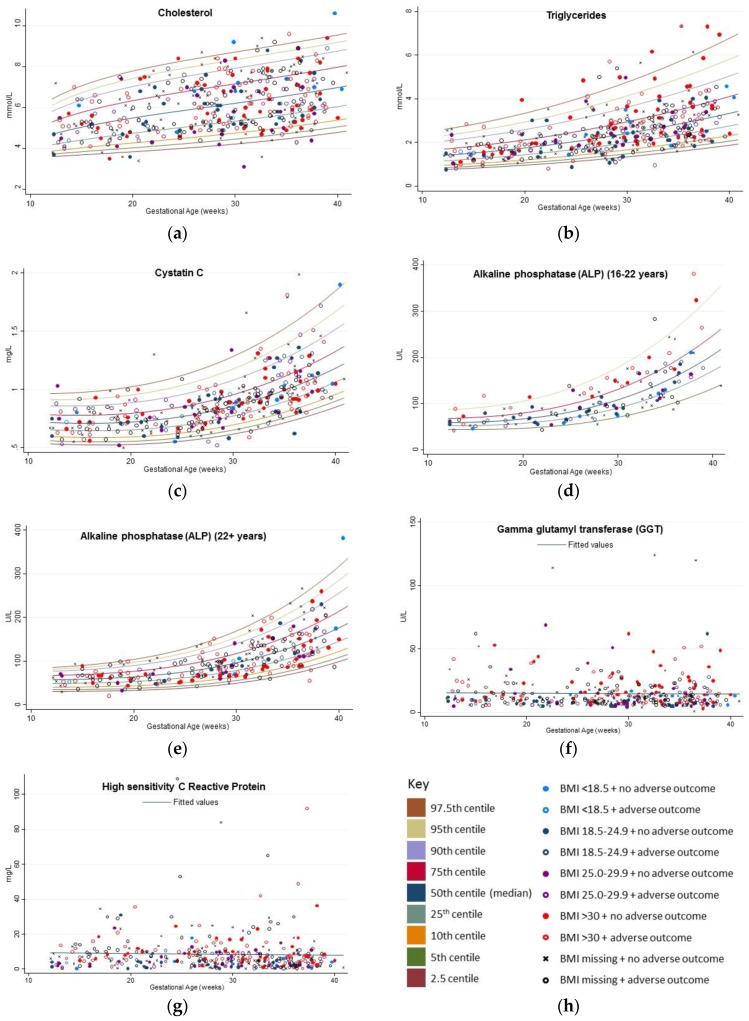
Non-fasting percentile ranges: (**a**) Cholesterol (*n* = 398 observations); (**b**) triglycerides (*n* = 397 observations); (**c**) Cystatin C (*n* = 348 observations); (**d**) ALP (16 years to less than 22 years) (*n* = 132); (**e**) ALP (22 years and older) (*n* = 263 observations); (**f**) GGT (*n* = 397 observations); (**g**) hs-CRP (*n* = 382 observations). (**h**) Key designating colors and symbols for centile bands, BMI categories and status of adverse outcomes related to pregnancy. Adverse outcomes related to pregnancy include gestational hypertension, preeclampsia, gestational diabetes, proteinuria, albuminuria, large for gestational age and small for gestational age babies.

Non-linear relationships were found for total cholesterol (MEN model), triglycerides (MEN model), ALP (both those aged less than 22 years and those aged 22 years and older) (EN model) and cyst-C (MEN model) (see Figure 1a–e and Table S1). Results for ALP (16–22 years) should be treated with caution, as a goodness of fit test could not be successfully fitted, possibly due to insufficient observations available. Ranges for hsCRP and GGT did not change over the course of the pregnancy (see Figure 1f,g). Cholesterol showed a tendency to slowly increase over the course of the pregnancy, (see Figure 1a). Triglycerides showed a greater increase towards the latter stages of the pregnancy (see Figure 1b), and Cyst-C increased particularly after week 30 of gestation (see Figure 1c). ALP showed exponential growth towards the end of the pregnancy in both those aged under and over 22 years (see Figure 1d,e). A summarization of changes over gestation time in percentile bands is shown in Tables S2–S6. The percentiles for GGT and hs-CRP are shown in Table S7.

With regard to BMI status, those categorized as overweight or obese according to their pre-pregnancy weight are over-represented above the 90th percentile for both triglycerides and ALP for 16–22 year olds, compared to those that are underweight or of healthy weight status (see Figure 1b,d,h). Additionally, those with higher GGT values are more commonly those categorized as overweight or obese. No clear patterns are discernible in relation to adverse pregnancy outcomes and the biomarkers of interest (see Figure 1).

## 4. Discussion

Percentile bands for serum lipids and inflammatory factors related to CVD health have been generated from the pregnancy data of 206 Indigenous Australian women, and show trajectories related to gestational age for total cholesterol, triglycerides, cyst-C and ALP. GGT and hs-CRP remained unchanged across gestation. The bands demonstrate the various percentiles where values fall in relation to these factors related to CVD health at according to gestation length and highlights values at the upper and lower extremities. These percentile ranges provide a guideline as to values that may be considered consistent with this cohort of Indigenous women at the mid to late stages of their pregnancy.

Many factors can influence the levels of lipids and inflammation markers described here during pregnancy. These broadly encompass changes related to fasting status, genetic or ethnic diversity, presence of possible or underlying disease states, and the effects of environmental and social factors, including lifestyle, dietary patterns and body composition [22]. The change from a fasting to a non-fasting state is one that has an impact on serum lipids relatively quickly. Triglycerides peak within 3–4 h post-prandial, with troughs occurring after this time, with a maximum mean change of 0.3 mmol/L to be expected [23]. Total cholesterol, after adjustment for albumin levels will decrease approximately −0.2 mmol/L 0–2 h after the last meal [23]. Women were non-fasting in this study, and it is unknown when and what foods and beverages were consumed prior to sample collection. 

Ethnic and genetic diversity extends beyond common anthropometric differences, and can be seen in a number of inflammatory and lipid markers [24,25]. The risk associated with these biomarkers can vary between ethnicities [25]. The majority of women in this study identified as Indigenous (83%), with the remaining 29 identifying as carrying an Indigenous child. Ethnicity includes social, cultural and traditional norms; it cannot be assumed that the women who did not personally identify as Indigenous were not experiencing the cultural life associated with the Indigenous status of their baby.

BMI—and intra-abdominal fat, more specifically—can increase the levels of serum triglycerides and total cholesterol [26]. The BMI status of the cohort presented here is comparable to underweight and healthy weight proportions reported by the Australian Bureau of Statistics (ABS) in the 2011-12 Australian Health Survey [27]. The ABS reported 7.3% of Indigenous women aged 18–24 years were categorized as underweight, with 34.1%, 28.2% and 30.4% being categorized as healthy, overweight and obese, respectively [27]. For the 25–34 years category, values of 6.5%, 29.2%, 26.8% and 37.5% were provided for underweight, healthy weight, overweight and obese categories, respectively [27]. There were fewer women categorized here as overweight (21.1%) compared to the national data, with greater proportions being seen in the obese category (45.6%). It is possible that this level of obesity contributed to higher triglyceride values. [23]. Higher values for triglycerides may also be due to the presence of gestational diabetes mellitus (GDM) [10]. A meta-analysis by Ryckman et al. showed levels for triglycerides to be elevated by approximately 0.3 mmol/L through the second and third trimesters in those diagnosed with GDM. It is possible that those contributing to the higher percentiles here had either diagnosed or undiagnosed GDM. Alternatively, higher triglycerides may be due to dietary intake factors.

Dietary patterns prior to and during pregnancy have also been shown to influence levels of serum lipids [28,29]. In particular, dietary patterns prior to pregnancy with relatively high intakes of discretionary foods such as cakes, snacks and candies, have been demonstrated to raise triglyceride levels, particularly in the second trimester [28]. Conversely, dietary patterns consistent with recommendations for following a DASH-type diet have shown negative associations with serum triglycerides [29]. DASH-type diets are characterized by high intakes of plant-based foods such as fruits and vegetables, and limited intakes of saturated fats and discretionary food choices [30]. Common over-the-counter medications such as vitamin and mineral supplements may also significantly affect serum lipids and inflammatory markers [31,32,33]. Post-partum dietary data for this cohort has been published elsewhere [34], but it is beyond the scope of this analysis to include the effects of dietary intakes and patterns here. 

When considering total cholesterol values in percentiles by trimester presented by Piechota et al. [9], the cholesterol values of the Gomeroi gaaynggal cohort are slightly lower. The 50th centile for a Polish pregnant cohort in the second and third trimester by Piechota et al. were 6.32 and 7.38 mmol/L, respectively [9]. Here, the value ranges were 4.68–5.79 mmol/L in the 12–24th weeks and 5.86–6.98 mmol/L in weeks 25–40. It is possible that some of this difference is due to the reduction in total cholesterol in some stages of the post-prandial state [23], although this does not explain the entire difference. The total cholesterol values for this Indigenous cohort are more similar to the mean fasting values of 6.85 ± 1.45 mmol/L for the 37–40 week of gestation provided by a Spanish cohort [35], although percentiles are not available to compare values in the upper and lower boundaries. 

With regard to comparability to national Indigenous data for smoking status during pregnancy, fewer women in this cohort reported smoking (34.1%) compared with rates published in 2014 (41%) [27]. Smoking has been shown to reduce levels of high-density lipoproteins [36], thereby lowering total cholesterol values. The lipoprotein fractions in this study are unknown. 

Cyst-C has been used as a measure of glomerular filtration rate [18], but may also indicate preeclampsia [37]. The glomerular filtration rate increases during normal pregnancy conditions, and cyst-C has been demonstrated to increase primarily in the third trimester [18]. Similar changes in cyst-C can be seen here, with higher levels being seen after week 30. Comparison of women with and without preeclampsia have shown small detectable differences in levels of cyst-C (1.43 ± 0.29 and 1.07 ± 0.21, respectively) [37]. However, this may be more predictive of preeclampsia in women of normal BMI than in those in the obese category [38].

hs-CRP, ALP and GGT may rise in conjunction with other infections or disease states, and may also be reflective of obesity or smoking status, or the pregnancy state itself. However, they have also been associated with adverse outcomes related to CVD [15,16,39]. Interestingly, there may be ethnic differences in hs-CRP during pregnancy [40]. The median value for hs-CRP in the Gomeroi gaaynggal cohort is 6.00 mg/L, comparable to 5.8 ± 4.2 mg/L (mean ± SD) reported by Ertas et al. in a cohort of 115 healthy women residing in Turkey [41]. It is interesting to note that Ertas et al. reported hs-CRP values of 9.6 ± 7.1 mg/L for women with mild preeclampsia and 23.4 ± 16.5 mg/L for women with severe preeclampsia [41]. These values are comparable with the 75th and 95th percentiles presented in this study, although the values presented by Ertas et al. were derived from a non-obese population. The population here has a high level of obesity, and this is known to play a role in increasing hs-CRP [41,42].

The increases in ALP are predominantly due to increases in placental ALP, one of four tissue specific forms [43]. Reference values from smaller cohort of 52 Swedish women provides upper and lower ranges for 17–24 weeks gestation of 39–105 U/L, 28–31 weeks of 52.5–118.8 U/L and 34–38 weeks of 87.6–228.6 U/L [44]. This corresponds with values presented in this cohort in the lower percentile ranges of approximately the 5th centile, and ranges in the upper limits between the 75th and 95th. Similarities were also seen in values presented for GGT; a lower limit of 5.4 U/L was given for weeks 17–24, 28–31 and 34–38, with upper limits ranging between 21.6 and 24.6 U/L [44]. As with the ALP, lower values correspond to the 5th–10th centile here, with upper values equating to the 75th–90th centiles. However, GGT is influenced by medication, and the medication status of the women in this study is unknown. 

One of the strengths of the design is the pragmatic approach taken, as women who went on to develop hypertension or preeclampsia were not excluded from the analysis. This means that values that may reflect abnormal values due to gestational diabetes, preeclampsia or other sub-clinical disease states are included and form part of the underlying data and, therefore, the percentile ranges. Also, whilst the sample size may be small compared to other Caucasian pregnancy cohorts, the data presented here represents values from one of the largest Indigenous pregnancy cohorts in Australia, with the population being relatively comparable to women in similar age groups in national Indigenous data. 

The method used to develop the reference percentiles used fractional polynomials, one of the few methods described by the World Health Organization as being suitable for developing reference ranges [45]. Whilst the Bhattacharya method has been used in the development of other serum-based reference ranges, it requires large amounts of data, and hence would have been unsuitable, given the sample size here [46].

Limitations include the varying length of time between when blood was collected and when it was centrifuged and analyzed. It is possible that the samples sustained changes in temperature, although they were stored in an insulated container with ice bricks prior to analysis. However, the serum lipids and inflammation factors presented here are unlikely to be significantly affected by either the variations in storage temperatures or the time taken until analysis [47,48]. More significantly, no data was collected as to medication usage that may have affected the serum lipids or inflammatory factors. Collection of data relating to medication usage during pregnancy is recommended for the development of similar pragmatic reference intervals.

## 5. Conclusions

This research will serve as a base for future reference ranges specific to Indigenous Australian women during pregnancy. Further development of CVD-related reference ranges for lipids and inflammatory factors for pregnant Indigenous Australian women would include comparing women in the higher and lower percentile ranges to adverse post-partum outcomes to either mother or child, or, conversely, those in the 25th–75th percentiles to normal post-partum outcomes. Also, inclusion of pregnant Indigenous women from more remote communities would more closely represent Indigenous women in Australia. Overall, this study will make a contribution towards improving the monitoring of biochemical markers in Indigenous women during this crucial health stage.

## Figures and Tables

**Table 1:** Demographic and health characteristics of women at the time of their pregnancy.

Demographic & Health Characteristic	Number of Pregnancies with Data (*n* = 206)	Mean (SD ^1^)	Median (IQR ^2^)
Age (at time of consent) (years)	(206)	25.5 (6.1)	24.3 (21.0–29.0)
Self-reported pre-pregnancy weight (kg)	(124)	79.1 (23.9)	76.5 (60.5–92.1)
BMI ^3^ based on pre-pregnancy weight (kg·m^−2^)	(114)	30.0 (8.4)	28.8 (22.9–36.6)
		n	(%)
BMI: underweight (<18.5)		7	(6.1%)
BMI: normal (18.5–24.99)		31	(27.2%)
BMI: overweight (25–29.99)		24	(21.1%)
BMI: obese (30 or higher)		52	(45.6%)
Reported smoking during pregnancy	(185)	63	(34.1%)
Self-reported diabetes status at consent ^4^	(117)	7	(6.1%)
(type 1)	-	3	(2.6%)
(type 2)	-	4	(3.4%)
Developed gestational diabetes in prior pregnancy	(180)	18	(8.7%)
Self-reported hypertensive at consent ^4^	(116)	6	(5.2%)
(don’t know/unsure)	-	4	(3.5%)
Developed gestational hypertension in prior pregnancy	(185)	11	(5.3%)
Developed preeclampsia in prior pregnancy	(184)	14	(6.8%)
Self-reported IHD ^4,5^ status at consent	(113)	0	(0%)
(don’t know/unsure)	-	3	(2.7%)

^1^ SD: standard deviation; ^2^ IQR: Interquartile range; ^3^ BMI: Body mass index; ^4^ Data available only after July, 2015; ^5^ IHD: Ischaemic heart disease.

## Supplementary Materials

The following are available online at http://www.mdpi.com/2227-9032/5/4/72/s1, Table S1: Distributions, fractional polynomial terms and goodness of fit values used in creating of reference ranges, Table S2: Non-fasting total cholesterol percentiles for Indigenous Australian women during pregnancy, Table S3: Non-fasting triglycerides percentiles for Indigenous Australian women during pregnancy, Table S4: Non-fasting cystatin C percentiles for Indigenous women during pregnancy, Table S5: Non-fasting ALP percentiles for Indigenous Australian women during pregnancy aged 16 to less than 22 years, Table S6: Non-fasting ALP1 percentiles for Indigenous Australian women during pregnancy aged 22 years and older, Table S7: Percentile bands for hs-CRP and GGT during pregnancy for Indigenous Australian women.
