# Supplementary material for: Reference Intervals for Non-Fasting CVD Lipids and Inflammation Markers in Pregnant Indigenous Australian Women
Source: Healthcare (Basel). 2017 Oct 14;5(4):72. doi: 10.3390/healthcare5040072 (PMC5746706; doi:10.3390/healthcare5040072)
Supplement: Supplementary file 1 [file healthcare-05-00072-s001.pdf]

Supplementary Materials: Reference Intervals for Non-Fasting CVD Lipids and Inflammation Markers in Pregnant Indigenous Australian Women

Tracy L. Schumacher, Christopher Oldmeadow, Don Clausen, Loretta Weatherall, Lyniece Keogh, Kirsty G. Pringle and Kym M. Rae

Table S 1: Distributions, fractional polynomial terms and goodness of fit values used in creating of reference ranges.

CVD factor	Distribution	Fractional polynomial terms				Goodness of fit
		M		S	G	(p)
Cholesterol	men	-0.5	3	-1	0	0.362
Triglycerides	men	-2	2	2	0	0.366
Cystatin-C	men	2	2	3	0	0.358
ALP (16-22 years)	en	3	3	3	0	0.583
ALP (22+ years)	en	2	3	3	0	Not available[1]

[1] Insufficient degrees of freedom for test of means. Results for the ranges provided for ALP 16-22 years should be treated with caution.

Table S 2. Non-fasting total cholesterol [1] percentiles for Indigenous Australian women during pregnancy

Gestation week	2.5th	5th	10th	25th	50th	75th	90th	95th	97.5th
12	3.60	3.72	3.87	4.19	4.68	5.26	5.78	6.09	6.38
13	3.63	3.76	3.93	4.27	4.81	5.45	6.00	6.34	6.64
14	3.65	3.80	3.98	4.35	4.93	5.61	6.19	6.55	6.86
15	3.68	3.84	4.04	4.43	5.04	5.76	6.37	6.73	7.06
16	3.71	3.88	4.09	4.50	5.14	5.89	6.52	6.90	7.23
17	3.74	3.92	4.13	4.57	5.24	6.01	6.66	7.05	7.39
18	3.77	3.95	4.18	4.63	5.33	6.13	6.79	7.19	7.53
19	3.81	3.99	4.23	4.70	5.41	6.23	6.91	7.31	7.66
20	3.84	4.03	4.27	4.76	5.49	6.33	7.02	7.43	7.78
21	3.87	4.07	4.32	4.82	5.57	6.43	7.13	7.54	7.90
22	3.90	4.11	4.37	4.87	5.65	6.52	7.23	7.64	8.00
23	3.94	4.15	4.41	4.93	5.72	6.60	7.32	7.74	8.10
24	3.97	4.19	4.46	4.99	5.79	6.69	7.41	7.83	8.20
25	4.01	4.23	4.50	5.05	5.86	6.77	7.50	7.92	8.29
26	4.05	4.27	4.55	5.10	5.93	6.85	7.59	8.01	8.38
27	4.09	4.31	4.60	5.16	6.00	6.93	7.67	8.10	8.46
28	4.13	4.36	4.65	5.22	6.07	7.01	7.75	8.18	8.55
29	4.17	4.41	4.70	5.28	6.14	7.08	7.83	8.26	8.63
30	4.22	4.45	4.76	5.34	6.21	7.16	7.91	8.34	8.71
31	4.26	4.50	4.81	5.40	6.28	7.24	8.00	8.43	8.79
32	4.31	4.56	4.87	5.47	6.35	7.32	8.08	8.51	8.87
33	4.36	4.61	4.92	5.53	6.42	7.39	8.16	8.59	8.95
34	4.42	4.67	4.98	5.60	6.50	7.47	8.24	8.67	9.04
35	4.47	4.73	5.05	5.66	6.57	7.55	8.32	8.75	9.12
36	4.53	4.79	5.11	5.73	6.65	7.64	8.4	8.84	9.20
37	4.59	4.85	5.18	5.81	6.73	7.72	8.49	8.92	9.29
38	4.65	4.92	5.25	5.88	6.81	7.80	8.58	9.01	9.37
39	4.72	4.98	5.32	5.96	6.89	7.89	8.66	9.10	9.46
40	4.79	5.06	5.39	6.04	6.98	7.98	8.75	9.19	9.55

[1]reference range for non-pregnant women: <5.5mmol/L **[21]**

Table S 3. Non-fasting triglycerides[1] percentiles for Indigenous Australian women during pregnancy

Gestation week	2.5th	5th	10th	25th	50th	75th	90th	95th	97.5th
12	0.75	0.84	0.95	1.15	1.40	1.70	2.05	2.32	2.61
13	0.77	0.86	0.97	1.18	1.43	1.74	2.10	2.38	2.68
14	0.79	0.89	1.00	1.21	1.47	1.78	2.16	2.45	2.76
15	0.82	0.91	1.03	1.24	1.51	1.83	2.22	2.52	2.85
16	0.84	0.94	1.06	1.28	1.55	1.89	2.29	2.60	2.94
17	0.87	0.97	1.09	1.31	1.59	1.94	2.36	2.68	3.03
18	0.89	1.00	1.12	1.35	1.64	2.00	2.43	2.76	3.14
19	0.92	1.03	1.16	1.39	1.69	2.06	2.50	2.85	3.24
20	0.95	1.06	1.19	1.44	1.74	2.12	2.58	2.95	3.35
21	0.98	1.10	1.23	1.48	1.80	2.19	2.67	3.05	3.47
22	1.02	1.13	1.27	1.53	1.86	2.26	2.76	3.15	3.59
23	1.05	1.17	1.31	1.58	1.92	2.34	2.85	3.26	3.72
24	1.09	1.21	1.36	1.63	1.98	2.41	2.95	3.37	3.85
25	1.12	1.25	1.40	1.69	2.05	2.49	3.05	3.49	3.99
26	1.16	1.29	1.45	1.74	2.11	2.58	3.15	3.61	4.14
27	1.20	1.34	1.50	1.80	2.19	2.67	3.26	3.74	4.29
28	1.24	1.38	1.55	1.86	2.26	2.76	3.37	3.87	4.44
29	1.29	1.43	1.60	1.92	2.34	2.85	3.49	4.01	4.61
30	1.33	1.48	1.66	1.99	2.41	2.95	3.61	4.15	4.77
31	1.38	1.53	1.71	2.06	2.5	3.05	3.73	4.29	4.95
32	1.42	1.58	1.77	2.13	2.58	3.15	3.86	4.45	5.13
33	1.47	1.63	1.83	2.20	2.67	3.26	4.00	4.60	5.31
34	1.52	1.69	1.89	2.27	2.76	3.37	4.13	4.76	5.50
35	1.58	1.75	1.96	2.35	2.85	3.48	4.27	4.93	5.70
36	1.63	1.81	2.02	2.43	2.94	3.60	4.42	5.10	5.90
37	1.68	1.87	2.09	2.51	3.04	3.72	4.57	5.27	6.11
38	1.74	1.93	2.16	2.59	3.14	3.84	4.72	5.45	6.33
39	1.80	1.99	2.23	2.67	3.24	3.97	4.88	5.64	6.55
40	1.86	2.06	2.30	2.76	3.35	4.10	5.04	5.83	6.78

[1]reference range for non-pregnant women: <2.00mmol/L **[21]**

Table S 4. Non-fasting Cystatin C[1] percentiles for Indigenous Australian women during pregnancy

Gestation week	2.5th	5th	10th	25th	50th	75th	90th	95th	97.5th
12	0.53	0.56	0.60	0.65	0.72	0.78	0.86	0.91	0.97
13	0.53	0.56	0.59	0.65	0.71	0.78	0.86	0.91	0.97
14	0.53	0.56	0.59	0.65	0.71	0.78	0.86	0.91	0.97
15	0.52	0.55	0.59	0.65	0.71	0.78	0.86	0.91	0.97
16	0.52	0.55	0.59	0.64	0.71	0.78	0.86	0.92	0.98
17	0.52	0.55	0.59	0.64	0.71	0.78	0.86	0.92	0.98
18	0.52	0.55	0.59	0.65	0.71	0.78	0.87	0.93	0.99
19	0.52	0.55	0.59	0.65	0.71	0.79	0.87	0.94	1.00
20	0.52	0.56	0.59	0.65	0.72	0.79	0.88	0.95	1.02
21	0.53	0.56	0.60	0.66	0.73	0.80	0.89	0.96	1.03
22	0.53	0.56	0.60	0.66	0.73	0.81	0.91	0.98	1.05
23	0.54	0.57	0.61	0.67	0.74	0.82	0.92	0.99	1.07
24	0.55	0.58	0.62	0.68	0.75	0.84	0.94	1.01	1.09
25	0.55	0.59	0.63	0.69	0.77	0.85	0.95	1.03	1.11
26	0.56	0.60	0.64	0.70	0.78	0.87	0.97	1.05	1.14
27	0.57	0.61	0.65	0.72	0.80	0.89	1.00	1.08	1.17
28	0.59	0.62	0.66	0.73	0.81	0.91	1.02	1.11	1.20
29	0.60	0.64	0.68	0.75	0.83	0.93	1.05	1.14	1.24
30	0.62	0.65	0.70	0.77	0.86	0.95	1.08	1.17	1.28
31	0.63	0.67	0.71	0.79	0.88	0.98	1.11	1.21	1.32
32	0.65	0.69	0.73	0.81	0.90	1.01	1.14	1.25	1.37
33	0.67	0.71	0.76	0.84	0.93	1.04	1.18	1.29	1.42
34	0.69	0.73	0.78	0.86	0.96	1.08	1.22	1.34	1.47
35	0.71	0.76	0.81	0.89	0.99	1.11	1.26	1.39	1.53
36	0.74	0.78	0.83	0.92	1.03	1.15	1.31	1.44	1.59
37	0.76	0.81	0.86	0.96	1.06	1.19	1.36	1.49	1.65
38	0.79	0.84	0.89	0.99	1.10	1.23	1.41	1.55	1.72
39	0.82	0.87	0.93	1.02	1.14	1.28	1.46	1.62	1.79
40	0.85	0.90	0.96	1.06	1.18	1.33	1.52	1.68	1.87

[1]reference range for non-pregnant women: <2.00mmol/L **[21]**

Table S 5. Non-fasting ALP[1] percentiles for Indigenous Australian women during pregnancy aged 16 to less than 22 years

Gestation week	2.5th	5th	10th	25th	50th	75th	90th	95th	97.5th
12	42.40	44.61	47.37	52.56	59.49	68.28	78.88	87.36	97.06
13	42.15	44.44	47.27	52.62	59.75	68.79	79.68	88.39	98.32
14	42.00	44.36	47.29	52.82	60.18	69.51	80.74	89.72	99.94
15	41.96	44.40	47.44	53.17	60.80	70.47	82.10	91.39	101.95
16	42.03	44.58	47.74	53.70	61.64	71.69	83.78	93.42	104.38
17	42.25	44.91	48.21	54.43	62.72	73.21	85.81	95.86	107.26
18	42.61	45.40	48.86	55.38	64.06	75.04	88.23	98.73	110.64
19	43.15	46.08	49.72	56.57	65.69	77.22	91.06	102.07	114.55
20	43.87	46.96	50.80	58.01	67.62	79.76	94.32	105.91	119.02
21	44.80	48.06	52.11	59.73	69.88	82.69	98.06	110.28	124.09
22	45.94	49.40	53.68	61.75	72.48	86.04	102.3	115.21	129.79
23	47.32	50.98	55.53	64.08	75.47	89.84	107.06	120.73	136.17
24	48.95	52.84	57.67	66.76	78.85	94.11	112.38	126.88	143.24
25	50.84	54.98	60.12	69.78	82.64	98.87	118.29	133.69	151.06
26	53.02	57.42	62.90	73.19	86.88	104.15	124.82	141.20	159.65
27	55.49	60.19	66.02	76.99	91.58	109.98	131.99	149.42	169.05
28	58.28	63.29	69.51	81.21	96.77	116.38	139.84	158.4	179.30
29	61.39	66.74	73.38	85.86	102.46	123.38	148.39	168.17	190.43
30	64.86	70.56	77.65	90.97	108.68	131.00	157.67	178.76	202.48
31	68.68	74.77	82.34	96.56	115.46	139.27	167.72	190.21	215.48
32	72.88	79.39	87.46	102.64	122.81	148.22	178.56	202.53	229.46
33	77.48	84.42	93.04	109.23	130.76	157.87	190.22	215.78	244.47
34	82.49	89.89	99.09	116.37	139.32	168.24	202.73	229.97	260.54
35	87.92	95.82	105.62	124.05	148.53	179.36	216.13	245.15	277.70
36	93.79	102.22	112.67	132.31	158.41	191.26	230.43	261.34	295.98
37	100.13	109.10	120.24	141.17	168.97	203.96	245.67	278.57	315.43
38	106.93	116.49	128.35	150.64	180.24	217.49	261.88	296.88	336.09
39	114.23	124.40	137.02	160.74	192.24	231.87	279.09	316.31	357.97
40	122.03	132.85	146.28	171.50	205.00	247.13	297.32	336.87	381.13

[1]reference range for non-pregnant women: <35-140 U/L (16<22 yrs) **[21]**

Table S 6. Non-fasting ALP[1] percentiles for Indigenous Australian women during pregnancy aged 22 years and older

Gestation week	2.5th	5th	10th	25th	50th	75th	90th	95th	97.5th
12	30.64	34.92	39.89	48.29	57.76	67.38	76.16	81.48	86.14
13	30.86	35.10	40.05	48.47	58.07	67.92	77.03	82.59	87.48
14	31.07	35.29	40.24	48.73	58.48	68.61	78.06	83.88	89.04
15	31.30	35.53	40.50	49.08	59.03	69.46	79.29	85.39	90.83
16	31.57	35.82	40.84	49.55	59.73	70.49	80.74	87.15	92.89
17	31.89	36.18	41.28	50.15	60.59	71.73	82.43	89.18	95.26
18	32.28	36.64	41.83	50.90	61.64	73.20	84.40	91.51	97.96
19	32.77	37.21	42.51	51.81	62.90	74.92	86.66	94.17	101.03
20	33.37	37.91	43.33	52.90	64.38	76.91	89.25	97.20	104.49
21	34.10	38.76	44.33	54.21	66.11	79.20	92.19	100.63	108.40
22	34.98	39.77	45.51	55.73	68.12	81.81	95.52	104.47	112.78
23	36.02	40.96	46.90	57.50	70.41	84.78	99.26	108.78	117.66
24	37.25	42.36	48.52	59.53	73.01	88.11	103.43	113.58	123.09
25	38.68	43.98	50.37	61.85	75.96	91.84	108.08	118.9	129.11
26	40.33	45.84	52.5	64.47	79.26	96.00	113.23	124.78	135.74
27	42.23	47.96	54.90	67.42	82.94	100.60	118.90	131.26	143.03
28	44.39	50.36	57.62	70.72	87.03	105.68	125.14	138.36	151.02
29	46.83	53.07	60.65	74.39	91.54	111.27	131.97	146.12	159.75
30	49.58	56.10	64.04	78.45	96.51	117.39	139.42	154.58	169.27
31	52.65	59.48	67.80	82.93	101.96	124.06	147.53	163.77	179.60
32	56.06	63.21	71.94	87.85	107.92	131.32	156.33	173.73	190.79
33	59.84	67.34	76.50	93.23	114.40	139.20	165.85	184.51	202.90
34	64.01	71.87	81.49	99.09	121.43	147.72	176.12	196.12	215.95
35	68.58	76.84	86.94	105.46	129.05	156.90	187.18	208.62	230.00
36	73.59	82.26	92.88	112.37	137.27	166.79	199.05	222.04	245.08
37	79.05	88.15	99.31	119.84	146.12	177.41	211.79	236.42	261.26
38	84.98	94.54	106.27	127.88	155.63	188.79	225.41	251.80	278.56
39	91.41	101.45	113.78	136.54	165.82	200.95	239.95	268.22	297.05
40	98.36	108.91	121.87	145.82	176.73	213.94	255.45	285.72	316.76

[1]reference range for non-pregnant women: <30-110 U/L(22<120 yrs) **[21]**

Table S 7. Percentile bands for hs-CRP and GGT during pregnancy for Indigenous Australian women

Inflamm-atory Marker	Unit	2.5th	5th	10th	25th	50th	75th	90th	95th	97.5th
hsCRP[1]	(mg/L)	0.50	0.90	1.50	3.00	6.00	10.00	18.00	25.00	34.60
GGT[2]	(U/L)	5.0	5.0	6.0	8.0	11.0	16.0	26.0	37.0	52.0

1 Reference range for non-pregnant women: 1.00-3.00 mg/L.

2 Reference range for non-pregnant women: 9-36 U/L

© 2017 by the authors; licensee MDPI, Basel, Switzerland. This article is an open access article distributed under the terms and conditions of the Creative Commons by Attribution (CC-BY) license (http://creativecommons.org/licenses/by/4.0/).
